# Effect of pulsed methylprednisolone on disease severity, viral load and inflammation in patients with human T-lymphotropic virus type 1 associated myelopathy

**DOI:** 10.1186/1742-4690-12-S1-P96

**Published:** 2015-08-28

**Authors:** Kevin Buell, Aiysha Puri, Maria Antonietta Demontis, Charlotte L Short, Adine Adonis, Jana Haddow, Fabiola Martin, Divya Dhasmana, Graham P Taylor

**Affiliations:** 1 Section of Retrovirology and GU Medicine, Department of Medicine, Imperial College London, London, UK; 2 National Centre for Human Retrovirology, Imperial College Healthcare NHS Trust, London, UK; 3 Centre of Immunology and Infection, Hull York Medical School, Department of Biology, University of York, York, UK

## 

The efficacy of treatments used for patients with HTLV-1 associated myelopathy/tropical spastic paraperesis (HAM/TSP) is uncertain although corticosteroids are widely prescribed. The effect of pulsed IV methylprednisolone was retrospectively analysed in an open cohort of 26 patients. 1g IV methylprednisolone was infused on three consecutive days. The outcomes were pain, gait, urinary frequency and nocturia, a range of inflammatory markers and HTLV-1 proviral load. A plasma cytokine profile was conducted in nine patients and correlated to gait and pain. Significant improvements in pain and 10m timed walk were observed immediately after the 3^rd^ infusion and maintained, for pain, for up to six months. A shorter duration of disease was strongly correlated with improvement in the 10m timed walk (p=0.05) but not with the reduction in pain. Although baseline cytokine concentrations did not correlate to baseline pain or gait impairment a decrease in tumour necrosis factor-alpha (TNF-α) concentration after pulsed methylprednisolone was associated with improvements in both. Pulsed IV methylprednisolone significantly reduced pain in patients with HAM/TSP and was associated with a transient improvement in gait that may be related to a reduction in TNF-α concentration.

